# Oral mucositis: the hidden side of cancer therapy

**DOI:** 10.1186/s13046-020-01715-7

**Published:** 2020-10-07

**Authors:** Claudio Pulito, Antonio Cristaudo, Caterina La Porta, Stefano Zapperi, Giovanni Blandino, Aldo Morrone, Sabrina Strano

**Affiliations:** 1 Oncogenomic and Epigenetic Unit, IRCCS, Regina Elena National Cancer Institute, Rome, Italy; 2 STI/HIV Unit, San Gallicano Dermatological Institute IRCCS, Rome, Italy; 3 Center for Complexity and Biosystems, Department of Environmental Science and Policy, University of Milan, via Celoria 26, 20133 Milano, Italy; 4 CNR - Consiglio Nazionale delle Ricerche, Istituto di Biofisica, via Celoria 26, 20133 Milano, Italy; 5 Center for Complexity and Biosystems, Department of Physics, University of Milan, Via Celoria 16, 20133 Milano, Italy; 6 CNR - Consiglio Nazionale delle Ricerche, Istituto di Chimica della Materia Condensata e di Tecnologie per l’Energia, Via R. Cozzi 53, 20125 Milano, Italy; 7 Scientific Director Office, San Gallicano Institute, Rome, Italy; 8 SAFU Laboratory, Department of Research, Advanced Diagnostic, and Technological Innovation, IRCCS, Regina Elena National Cancer Institute, Via Elio Chianesi, 53, 00144 Rome, Italy

**Keywords:** Oral mucositis, HNC, Biomarker, Cytokine, Non-coding RNA, Quality of life

## Abstract

Inflammation response of epithelial mucosa to chemo- radiotherapy cytotoxic effects leads to mucositis, a painful side effect of antineoplastic treatments. About 40% of the patients treated with chemotherapy develop mucositis; this percentage rises to about 90% for head and neck cancer patients (HNC) treated with both chemo- and radiotherapy. 19% of the latter will be hospitalized and will experience a delay in antineoplastic treatment for high-grade mucositis management, resulting in a reduction of the quality of life, a worse prognosis and an increase in patient management costs. Currently, several interventions and prevention guidelines are available, but their effectiveness is uncertain. This review comprehensively describes mucositis, debating the impact of standard chemo-radiotherapy and targeted therapy on mucositis development and pointing out the limits and the benefits of current mucositis treatment strategies and assessment guidelines. Moreover, the review critically examines the feasibility of the existing biomarkers to predict patient risk of developing oral mucositis and their role in early diagnosis. Despite the expression levels of some proteins involved in the inflammation response, such as TNF-α or IL-1β, partially correlate with mucositis process, their presence does not exclude others mucositis-independent inflammation events. This strongly suggests the need to discover biomarkers that specifically feature mucositis process development. Non-coding RNAs might hold this potential.

## Background

The advent of chemotherapy (CT) in 1940 led to a dramatic increase in mucositis adverse events, generically identified as stomatitis. The lack of efficacious therapies and of prevention guidelines for stomatitis consistently decreased patient quality of life and prognosis. Only sixty years later the complex mechanisms underlying mucositis pathogenesis were discovered and in 2007 the term mucositis was adopted to describe lesions associated to chemo- and/or radiotherapy (RT) cytotoxic effects.

Mucositis affects all gastro-intestinal tract and oral cavity inducing patient pain, inability to eat, weight loss and local infection. Furthermore, patients affected by a high-grade mucositis have to reduce chemotherapy regimen with delayed cancer treatment and worse prognosis. About 30–40% of cancer patients treated with chemotherapy develops mucositis, this percentage rises to 60–85% for patients undergoing to an hematopoietic stem cell transplantation (HSCT) and to almost 90% for head and neck cancer (HNC) patients treated with radio- plus chemotherapy [1]. Mucositis development leans not only on anticancer regimen, doses and number of cycles, but, also, on patients characteristics. Female patients, indeed, have a greater risk of developing severe mucositis when treated with 5-Fluoruracil (5-FU) [2], likewise to patients with a deficiency in the dihydropyrimidine dehydrogenase, a critical enzyme for 5-FU catabolism [3]. Conversely, patients affected by an aberrant epithelial proliferation, as psoriasis, exhibit a reduction in mucositis incidence [1]. In general old age, female gender, high bodyweight, a reduced clearance of drugs and genetic susceptibility are mucositis-related development risk factors.

Mucositis epidemiological data are still underestimated and contradictory. This adverse event, indeed, is often recorded only when patients develop a high-grade mucositis for which a clinical treatment is required. Moreover, there is not a standard scale to score its severity, thus making the disease staging and assessment rather difficult to compare. Currently, there are different scales to grade mucositis, whose parameters vary among them. The World Health Organization (WHO) scale for oral mucositis (OM) evaluation accounts for objective criteria, such as the presence of either erythema or ulceration. These are functional criteria based on the ability of the patient to eat. A quantitative scale that assesses ulceration dimension is used by the Oral Mucositis Assessment Scales (OMAS). The Eastern Cooperative Oncology Group (ECOG) mucositis scale is reported in the common toxicity criteria guide in which mucositis severity is differently classified based on the anatomic site of development. Similarly, the National Cancer Institute (NCI) provides in the Common Terminology Criteria for Adverse Event (CTCAE) mucositis severity measure scale based on anatomic site of development and on the kind of treatment, either chemo or radiotherapy [4].

### **Mucositis pathogenesis**

**Fig. 1:**
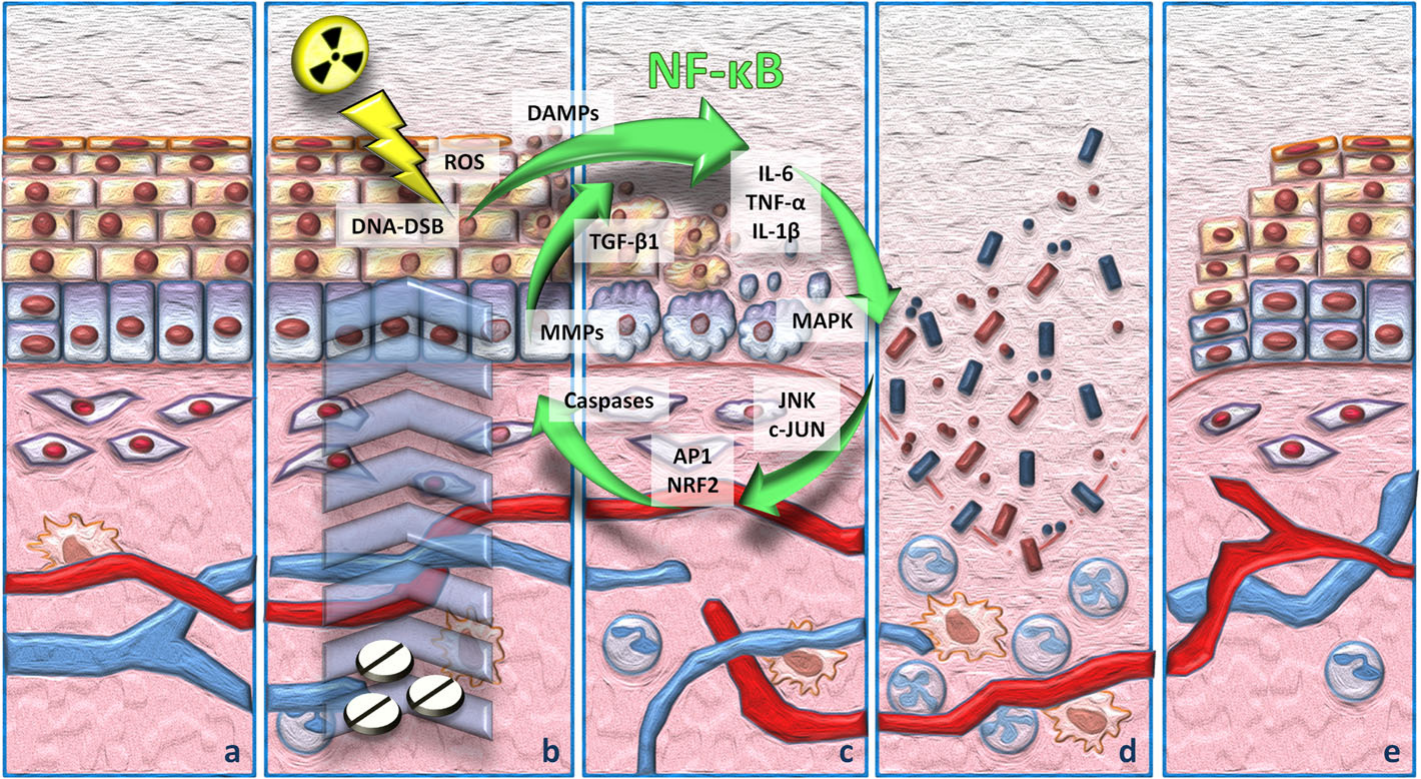
Mucositis pathobiology: (**a**) normal tissue; (**b**) initiation phase and primary injury response. Radio and chemotherapy-induced damages lead to an increase in DNA double strand brakes and ROS production with a consequent induction of cell apoptosis and DAMPS release. DAMPs and ROS signaling promote the NF-κB-mediated transcription of cytokines; (**c**) amplification of the injury signal. The effectors produced during the previous phase lead to an amplification of the injury signal. The released TNF-α initiates the activation of MAPK that sustains NF-κB activity. During this stage, the primary damage signaling is amplified through positive-feedback loop mechanisms. (**d)** ulceration. Breaks in the submucosa allows to microorganisms to invade this tissue district leading to mononuclear-infiltrating cells-mediated inflammation response; (**e**) tissue re-epithelialization. Stimuli from the submucosa extracellular matrix and mesenchyme promote the healing process

Mucositis development consists of a cascade of events that can be divided in five stages occurring consecutively and mechanistically linked (Fig. 1). The injury of mucosa membranes, named ***mucositis initiation*** phase, is caused by either radio- and/or chemotherapy. This stage occurs concurrently with chemo- or radiotherapy administration. Systemic chemotherapy and radiotherapy induce tissue damage causing reactive oxygen species (ROS) release, DNA damage thereby leading to cell death of the basal and suprabasal epithelial cells [5]. In particular, DNA strands breaks lead to the activation of the apoptotic process which is regulated by p53 activation and increased caspase 3 [6]. As a direct consequence of it, dead cells release endogenous damage-associated pattern molecules (DAMPs). This primary damage response characterizes the second stage of mucositis development (Fig. 1). During this stage cells of the injured mucosa promote the transcription of several genes involved in the mucositis process. In this molecular scenario, the nuclear factor-κB (NF-κB) represents the main transcriptional mediator modulating over 200 genes associated with pro-inflammatory cytokines (tumor necrosis factor α/TNF-α; interleukin-6/IL-6; interleukin-1β/IL-1β), cell adhesion molecules, stress responders and cytokine modulators [7, 8]. The presence of pro-inflammatory cytokines is, also, reported within the mucosa, where they seem to induce early damage of connective tissue and endothelium, as well as to inhibit tissue oxygenations and to favor epithelial basal cell death [5]. During this phase the activation of the immediate response genes, as well as the activation of c-JUN and the c-JUN aminoterminal kinase (JNK) takes place; thereby following the release of cell membrane bound molecules lead to activation of other transcription factors involved in the process [9]. Among them, the nuclear factor erythroid 2-related factor 2 (NRF2) is a basic leucin zipper protein that promotes the expression of antioxidant proteins as consequence of injury and inflammation process [10]. Moreover, anticancer treatment damages also fibroblasts, thus leading to the activation of protein-1 (AP1) and the consequent secretion of metalloproteinases (MMPs), such as MMP1 and MMP3 which degrade collagenous sub-epithelia matrix and disaggregate the epithelial basement membrane respectively [5, 11].

The effectors produced during the primary damage response lead to an amplification of the injury signal (Fig. 1). Concurrently with the activation of other pathways the primary damage is amplified through positive-feedback loop mechanisms. The released TNF-α, indeed, initiates the activation of the mitogen activated protein kinase (MAPK) on target cells and, in the same time, sustains NF-κB activity. During this stage, several damages impair the mucosa and sub mucosa structures. However, patients exhibit few symptoms and the mucosa does not reveal any macroscopic evidence of injury. MAPK signaling mediates caspase 3 activation and cell death through the activation of JNK that in turn, finely tunes AP1 transcriptional activity [9]. Moreover, the high levels of TNF-α activate sphingomyelinase increasing the pro-apoptotic signal mediated by the ceramide pathway and, together with IL-1β, modulate MMP1 and MMP3 activities [11, 12]. Besides, the injured keratinocytes release the transforming growth factor beta 1 (TGF-β1), that in turn, inhibits cell cycle, recruits leucocytes and sustains NF-κB activity, thus improving the damage-mediated signaling [13].

Clinical manifestations of mucositis are appreciable at the fourth stage of the inflammation process, the ulceration phase. During this stage, mucosa and sub mucosal integrity is compromised, patients complain of pain and may need caregivers management (Fig. 1). The presence of breaks in the submucosa, allows several microorganisms, symbiotic inhabitant of the healthy mucosa, to invade this tissue district leading to mononucler-infiltrating cells-mediated inflammation response, thus promoting new pro-inflammatory cytokines release that amplify expression of pro-apoptotic mediators and increase tissue damage [14, 15]. Patients, based on the duration and the extent of neutropenia, can develop bacteremia or septicemia, mainly caused, in OM, by streptococci and staphylococci [16]. Mucositis is an acute event that mostly self-resolves as the anticancer treatment ends. At this stage the healing process is activated, during which stimuli from the submucosa extracellular matrix and mesenchyme promote tissue re-epithelialization [5, 17].

### Role of “old” and “new” anticancer agents in mucositis development

Mucositis incidence and its severity depend on chemotherapy regimen, doses and treatment timing. Antimetabolites, platin-derived, taxanes, anthracyclines, irinotecan and alkylating agents can promote mucositis, whose severity and development site vary among the different drugs. Indeed, the antimetabolites drugs, S-1 and capecitabine are associated to a lower risk of mucositis development compared to the 5-FU treatment [18]. Conversely, when capecitabine is associated to irinotecan (XELIRI regimen), a topoisomerase 1 inhibitor, it induces higher gastro-intestinal mucositis (GIM) events compared to fluoruracil plus irinotecan combination (FOLFIRI) [19]. Nevertheless, patients treated with FOLFIRI regimen have a high probability to develop mucositis compared to patients treated with irinotecan plus oxaliplatin (FOLFOX) [19]. The role of irinotecan treatment in GIM development has been extensively studied so far. Irinotecan-mediated GIM clinical manifestation is characterized by two phases. Initially, treatment promotes the cholinergic syndrome and an early-onset diarrhea caused by an excess acetylcholine secretion due to the inhibition of acetyl cholinesterase. Afterwards, patients develop a late-onset diarrhea as a consequence of the irinotecan treatment that leads to mucin hyper-secretion, a reduction in goblet cell numbers and a general disaggregation of the gastrointestinal mucosa structure [20].

Cisplatin has been reported to induce OM through an indirect inhibition of mucin secretion, while it specifically damages the ileal mucosa rather than the remaining gastro intestinal tract [21]. GIM severity is higher in patients treated with cisplatin than in patients treated with other platinum-derived drugs, such as oxaliplatin and carboplatin [22].

Antineoplastic agents such as taxanes promote mucositis in a wide range of patients. However, only few of them develop a high grade mucositis; they generally present a mild or moderate event. Notably, docetaxel treatment is associated with a higher risk of mucositis development compared to paclitaxel [23].

The risk to develop mucositis rises when chemotherapy is associated to radiotherapy. Almost the 90% of HNC patients, indeed, develop mucositis when treated with chemo plus radiotherapy. Notably, HNC patients treated with cisplatin plus RT specifically develop oral mucositis [24]. In addition, patients receiving conventional RT fractions show a higher mucositis development risk compared to patients treated with high-dose single-fraction IMRT (5.1 versus 4.1-fold increased risk) [25]. During RT regimen, high-grade mucositis is more probably detectable either in patients characterized by a HPV/p16-negative status [26] or in those producing an elevated salivary cytokine, IL-6 and IL-1β, concentration [27]. Although the biological sequence of mucositis process is similar, radiotherapy treatment exerts its action on the target tissue within few seconds of exposure compared to CT. Irradiated patients complain GI burning pain just after the first week of treatment, while develop ulceration between the second and the third week of therapy. Unfortunately, RT-associated lesions persist for over six weeks after the latest session [28], affecting significantly the quality of life of the patients.

**Fig. 2:**
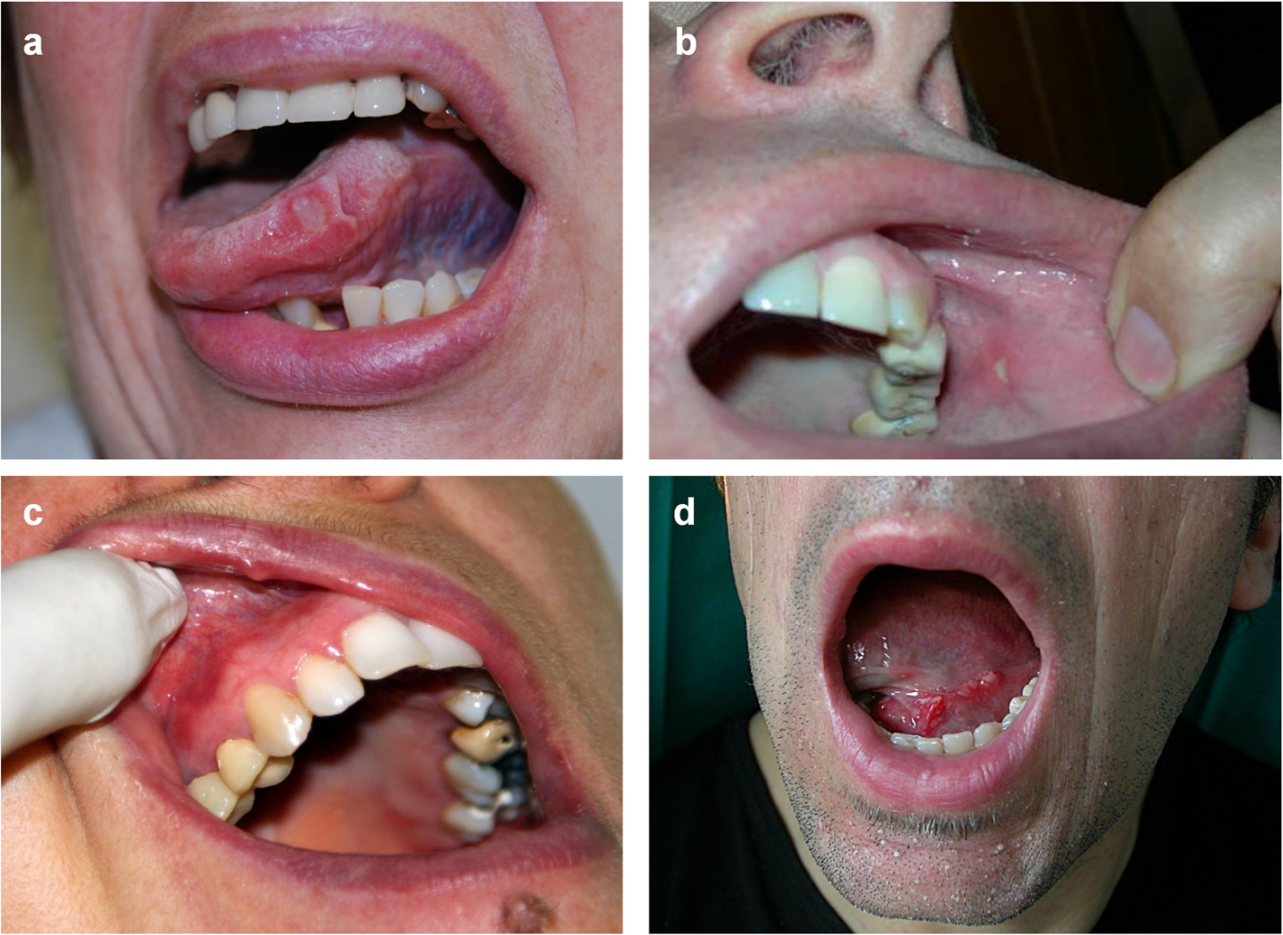
Representative images of mucositis induced by target therapies. (**a**-**b)** patchy ulcerations (aphtous ulcerations) induced by cetuximab, (**c**) erythema of the mucosa induced by temsirolimus and (**d**) ulcerations bleeding with minor trauma induced by everolimus

Beside standard chemo- and radiotherapy an increasing number of targeted agents is currently used in clinical practice for the management of different types of cancer. The target-specificity allows these new agents to have high efficacy and, at the same time, to promote less toxicity than standard CT. Nevertheless, clinicians face off with new toxicities whose characteristics vary accordingly to the administered target drugs. Among them, the mammalian target of rapamycin (mTOR)-inhibitor promotes a severe mucosal toxicity that differs from conventional oral mucositis. The mTOR-inhibitor-associated stomatitis (mIAS) is, indeed, smaller, relative shallow and extremely painful. Macroscopically, it presents a central necrotic area and a surrounding erythematous halo [29–31] (Fig. 2). It usually develops within five days after the first cycle of treatment and either improves or resolves spontaneously even during mTOR inhibitor regimen therapy [32]; however, it is often cause of a re-modulation of therapy dosage or, in presence of a high grade mIAS of treatment discontinuation. In the BOLERO-2 trial, indeed, the combination treatment of everolimus, an mTOR inhibitor, with examestane was limited by a high incidence of all-grade stomatitis in metastatic breast cancer women (67% of all-grade stomatitis, 33% grade 2 and 8% grade 3) [33]. Notably, patients treated with mTOR-inhibitors, such as everolimus, tenserolimus or ridaforolimus, exhibit an increased risk to develop oral stomatitis and enteritis [34]. mIAS-toxicity incidence changes accordingly to cancer types. Renal cell carcinoma (RCC) patients, in effect, present a lower risk to develop mIAS than astrocytoma, gastric and breast cancer patients [33]. This is also due to the characteristics of the drug that is associated to the mTOR-inhibitor that vary among the different cancer types, based on the clinical guidelines.

The epidermal growth factor (EGFR) inhibitors-associated mucosal lesions occur in 15% of treated patients [35]. Clinically they appear as limited lesions characterized by a moderate erythema, sometimes non dissimilar to an aphthous like lesions [36] (Fig. 2). As for mIAS, the onset is concomitant with the first cycle of treatment, may potentially affect all the non-keratinized area and can resolve autonomously during treatment [36]. Less than 1% of patients treated in mono-therapy with anti-EGFR antibodies, cetuximab or panitunumab, or EGFR-tyrosine kinase inhibitor (TKi), erlotinib or gefinitib, develop a high grade mucosal lesion, that in a very few cases require a treatment ri-modulation or suspension [37–39]. Conversely, the incidence and the severity of the lesion increase in those patients treated with a multi targeted TKi, such as afatinib, lapatinib or dacomitinib [40–42]. Indeed these cancer treatments are, associated with a higher incidence of all-grade mucositis compared to other EGFR-TKi (40% vs. 15% of all-grade, 8.7 vs. 1% of grade ≥ 3) [43] or to anti-EGFR monoclonal antibodies therapy [32]. Although mono-therapy with anti EGFR antibodies or TKi causes few mucosal lesions, these drugs are often associated to cytotoxic agents that cause an increase in mucositis incidence and severity. Indeed, the relative risk to develop high-grade mucosal lesions (≥ 3) significantly rises when cetuximab or panitunumab are administrated concomitantly with cisplatin, 5-FU, FOLFIRI or FOLFOX [32]. Moreover, although adding cetuximab to RT does not change the risk to develop a mucositis in HNC patients compared to RT alone (about 60% of high grade ≥ 3) [44, 45], however, when it is combined with RT plus CT increases the risk of high grade mucositis generation compared to RT plus CT treatment [46, 47].

Mucositis associated to ado-trastuzumab emtansine (T-DM-1) treatment are referable to a mucosal telangectasia [48]. T-DM-1 regimen is currently approved for HER2+, a member of EGF receptors aberrantly expressed in some tumors, metastatic breast cancer treatment. This treatment promotes a mucosal vascular malformation leading, in the 30% of patients, to epixastaxis and GI or gynecological bleeding [48, 49].

Oral mucositis, specifically stomatitis characterized by aphthous ulcer, occurs in very few patients treated with cyclin-dependent kinase 4/6 inhibitors (CDK4/6) [50, 51], used as first- or second-line treatment for hormone positive/HER2 negative metastatic breast cancer. However, this regimen promotes GIM rather than OM. Abemaciclib, indeed, induces an early-onset GIM in the first cycles of treatment in about 80% of the patients [51, 52]. Notably, CDK4/6 inhibitors bind, also, cyclin D3 in GI epithelial cells, inhibiting their proliferation and, consequently, inducing mucosal injury [53].

The BRAF and v-raf murine sarcoma viral oncogene homolog B1 inhibitors, vemurafenib and dafrafenib, have been recently approved by the Food and Drug Administration (FDA) for treatment of metastatic melanoma harboring BRAF^V600^ mutation. Patients treated with one of these inhibitors develop asymptomatic hyperkeratotic lesions rising both in the keratinized and non-keratinized mucosa, including mucosal lesions characterized by a verrucous or papillomatous appearance rising in the tongue, labial mucosa and linea alba [54–56].

Non-specific stomatitis, characterized by oral mucosal hypersensitivity and associated with a moderate erythema or inflammation painful, is described as an adverse event of anti angiogenetic drug treatment [57]. The probability to develop a stomatitis event changes accordingly to the target drug administrated. Indeed, bevacizumab or ramucirumab two monoclonal antibodies directed versus the vascular endothelial growth factor receptor (VEGFR), rarely induce stomatitis. Conversely, 25% of patients treated with multi- kinase inhibitors (MKIs), such as sunitinib, sorafenib or cabozantinib, complain stomatitis within the first two months of therapy [57]. However, less than the 10% of them need drug dosage re-modulation, while only 1% discontinues the treatment [57].

Low grade stomatitis is also described, although in few cases, as immunotherapy-related adverse event (irAE) in patients treated with the anti-programmed death 1 (PD1), pembrolizumab and nivolumab, or anti-programmed death ligand 1 (PDL1) drug, atezolizumab and durvalumab [32]. Patients treated with an immuno-check point drug suffer of different grade of diarrhea often associated to abdominal pain, dehydration and constipation [58, 59]. Endoscopic analysis has revealed presence of colic mucosa with a mild inflammation and/or ulceration upon PD1 or PDL1 treatment, while the same appeared ulcerated and friable in patients treated with a cytotoxic T lymphocyte-associated antigen 4 (CTLA4) inhibitor [35, 60]. Between the 27–54% of patients treated with a CTLA4 inhibitor, indeed, complain GI toxicities that often determine either treatment re-modulation or discontinuation [61].

### Mucositis prevention

While a growing number of new anti cancer agents are currently in clinical practice, only few therapeutic options are available for mucositis prevention or treatment. Their effectiveness is still poor. Notably, palifermin, a recombinant human keratinocyte growth factor 1 (KGF-1), is the only drug approved both by FDA and the European Medical Agency (EMA) for OM prevention in patients undergoing high doses CT plus total body RT prior to HSCT [62]. Palifermin acts stimulating epithelial cells proliferation and differentiation, thereby promoting faster tissue regeneration following chemo- and/or radiotherapy-induced damages. Moreover, it has antioxidant and antiapoptotic activities together with an anti pro-inflammatory action. The efficacy of this drug in preventing OM was also tested in HNC patients. Two different studies have demonstrated that patients treated with palifermin showed a lower incidence of high grade (≥ 3) OM [63, 64], however the high cost and the concerns about the possibility that this drug might sustain cancer cells growth makes it unsuitable for OM management in HNC patients.

In the following sections, we will resume clinical and pre-clinical evidences on the effectiveness of some of the so far tested drugs for mucositis development prevention grouped accordingly to their mechanism of action. A comprehensive list of drugs is reported in Table 1.

**Table 1:** List of therapies under investigation for mucositis development prevention grouped accordingly to their mechanism of action

**Antioxidant agents**	**Characteristics**	**Mechanism of action**	**References**
Amifostine	Phosphorylated aminosulfhydryl compound	Promotes recruitment of ROS scavenger, reduces DNA strand breaks	[65, 66]
Glutamine	Amino acid	Exerts antioxidant activities promoting glutathione synthesis	[67–71]
Oral zinc supplement	Essential mineral	Prevents lipids peroxidation, replaces redox reactive metals, induces metallothionein synthesis	[72–77]
Vitamin E	Lipid soluble α-tocopherol	Prevents tissue damages caused by the ROS release	[78–80]
N-acetyl-cysteine	N-acetyl derivative of the natural amino acid L-cysteine	Exerts antioxidant activities promoting glutathione synthesis, myeloperoxidase activity, xanthine dehydrogenase and oxidase activity.	[81, 82]
GC4419	Synthetic manganese-based drug	Counteracts superoxide dismutase activity	[83]
**Inhibitors of inflammation and cytokines production**	**Characteristics**	**Mechanism of action**	**References**
***Turmeric***	Flowering plant belonging to Curcuma longa	Counteracts NF- κB activiy	[84]
***Clonidine lauriad mucoadhesive buccal tablets***	Tablets contain high concentrations of an anti-inflammatory active principle (clonidine)	Inhibits NF-κB activity and the downstream pro-inflammatory cytokines-mediated signal	[85]
***SMAD7 over expression***	Gene encoding the nuclear protein Smad7 that binds the E3 ubiquitin ligase SMURF2	Impairs TGF-β1 that NF-κB activities in mice model (K5.Smad7) irradiated	[86]
***Benzydamine hydrochloride rinses***	Indazole non-steroidal anti-inflammatory drug	Inhibits the activity and the production of pro-inflammatory cytokines, TNF-α and IL-1β	[87, 88]
***Pentoxifylline***	Xanthine derivative	Impairs NF-κB activity and inhibits TNF-α and IL-1β action	[89, 90]
***Dusquetide (SGX942)***	5-amino acid innate defence regulator (IDR) peptide	Modulates immune innate pro-inflammatory response	[91]
**Multi target natural agents**	**Characteristics**	**Mechanism of action**	**References**
Honey	Honey topical application	Attenuates burns and pressure wounds.	[92–95]
Manuka and Kanuka essential oils	Mix of essential oil from *Leptospermum scoparium and Kunzea ericoides*	Anti-inflammatory, analgesic and anti-micotic and -bacterial activities	[96]
Chinese traditional herbs	1-Extract of Indigowood root 2-Extract of *Rhodiola algida*	1-Anti-inflammatory and antiviral activities 2-Stimulates the immuno system	[97, 98]
Chamomile mouthwash	infusion of powdered flower in water	Anti-inflammatory, analgesic and anti-micotic and -bacterial activities	[99, 100]
Aloe vera gel	Juice of succulent plant species of the genus *Aloe*	Promotes wound healing	[101]
MF 5232 (Mucotrol®)	Oral poliherbal gel wafer	Analgesic, wound healing and anti oxidant properties	[102]
Traumeel S®	Homeophatic complex mouthwash	Unknown mechanism of action	[103]
**Physical intervention**	**Characteristics**	**Mechanism of action**	**Reference**
Low-levels laser therapy (LLLT)	Monochromatic laser at low intensity	Promotes regeneration of damaged-tissue	[73, 83]
Oral cryotherapy	ice chips, ice cubes, ice lollipops	Promotes local vasoconstriction, thus leading to a reduction exposure of mucosa to chemotherapy	[73, 81, 104]
Oral care	Standardized oral care and frequent oral cavity examination by oral care experts	Prevents infections	[105]
**Lactobacillus**	Probiotic	Preserves mucosal intestinal architecture	[106, 107]

#### Antioxidant agents

Mucositis development is a multistep process, accordingly a good therapeutic option should impinge concurrently on different key pathways involved in its pathobiology without affecting the anti neoplastic regimen efficacy. In this regard, ROS, as early drivers of mucosa damage, represent a potential target for the inhibition of mucositis development. Antioxidant agents, such as amifostine, have been found to partially prevent mucositis development during RT treatment, reducing DNA strand breaks and preserving salivary gland, endothelium and connective tissue integrity [65, 66]. However, the related adverse events and the intravenous administration limit amifostine use in the routine clinical practice.

The administration of other ROS-scavenger drugs such as glutamine [67–71], oral zinc supplement [72–75, 77], vitamin E [78–80] or N-acetyl-cysteine (NAC) [81] have provided contradictorily evidences of their effectiveness in mucositis prevention. Recently, the MASCC/ISOO panel of experts have suggested the oral administration of glutamine tablets for OM prevention in HNC patients treated with CT-RT therapy. Conversely they have recommended against the parental administration of glutamine for OM prevention in patients undergoing to HSCT regimen due to the higher mortality rate associated to this treatment [108].

A synthetic manganese-based drug, GC4419, is currently investigated in a phase II study for mucositis prevention in HNC patients treated with cisplatin and RT, based on its ability to inhibit ROS production counteracting superoxide dismutase (SOD) activity [83] (clinicaltrials.gov identifier: NCT02508389).

####  Inhibitors of inflammation and cytokines production

As previously mentioned, NF-κB represents the main transcriptional mediator of mucositis process. It directly modulates transcription of several pro-inflammatory cytokines (TNF-α; IL-6; IL-1β) involved in the RT and CT-mediated damage signaling amplification [7, 8]. NF-κB has been also shown to promote drug resistance mechanisms [109, 110]; thereby the impairment of its action could affect mucositis development and cancer progression, concurrently. In this regard, turmeric, a flowering plant belonging to *Curcuma longa* of the ginger family, has been found to reduce and delay OM severity in HNC patients by counteracting NF-κB activity upon RT-mediated tissue injury [84]. Likewise, clonidine lauriad mucoadhesive buccal tablets (Clonidine Lauriad®) administration has been found to reduce the percentage of HNC patients developing high-grade mucositis (45.3% clonidine + CRT arm vs. 60% placebo + CRT arm), through the direct inhibition of NF-κB activity and of the downstream pro-inflammatory cytokines-mediated signal [85]. Intriguingly, transgenic mice expressing high levels of Smad7 (K5.Smad7) in oral epithelia resulted more resistant to radiation-induced oral mucositis development than wild type mice [86]. Indeed, high Smad7 levels, impairing concurrently both TGF-β1 that NF-κB activities, have been found to inhibit damage-mediated inflammation and promote a quickly epithelia self renewal, thus impairing OM development [86]. However, data obtained from the above reported evidences are very preliminary; further studies are mandatory to provide solid evidences of turmeric and clonidine effectiveness in mucositis prevention, as well as to understand the consequence of Smad7 over-expression on tumor behavior.

Conversely, there is strong evidence supporting the use of benzydamine hydrochloride rinses for OM prevention in HNC patients undergoing to RT, but not for those treated with either CT or CT-RT. Notably, the anti-inflammatory action of benzydamine has been found to inhibit the activity and the production of several pro-inflammatory cytokines, thus to reduce frequency of severe mucositis development (43.6% in benzydamine arm vs. 78.6% placebo arm) and percentage of patients developing mucosa erythema or ulceration [87, 88]. Basing on these data, EMA and the Multinational Association of Supportive Care in Cancer and International Society of Oral Oncology (MASCC/ISOO) guidelines recommend the use of benzydamine rinses in HNC patients undergoing to moderate-dose RT (< 50 Gy) and suggested the use also for OM prevention in HNC patients who receive RT-CT [108]. Further studies are ongoing to determine the efficacy of benzydamine also in patients managed with high-dose RT.

As well as benzydamine, pentoxifylline acts as anti-inflammatory agent inhibiting TNF-α and IL-1β. It has been demonstrated to decrease OM development in mice undergoing to irradiation [89] and, in association with vitamin E, has been found reducing RT-induced OM severity in a small cohort of HNC patients [90].

Dusquetide, SGX942, is an immune defense regulator agent able to modulate immune innate pro-inflammatory response and the subsequently signaling amplification that result over-activated during mucositis development. A randomized phase II study has reported that intravenous duquetide administration in HNC patients treated with CDDP and RT significantly reduced OM duration and rate of infection compared to patients belonging to the placebo arm [91]. Phase III studies, enrolling large cohorts of patients, will definitively support the effectiveness of pentoxifylline and duquetide in OM prevention.

#### Natural agents

Compared to most of the above-described drugs, natural compounds can be administrated as dietary supplements. Accordingly, they are often well tolerated by patients and do not induce severe adverse events. Moreover, thanks to their chemical structure, they can concurrently impinge on several cellular signaling pathways thus affecting mucositis pathogenesis process at different levels, as well as negatively impacting on tumorigenic activities of cancer cells [109, 111, 112]. Actually, diverse natural products have been already tested and others are under investigation in active clinical trials. Among the natural agents tested so far, glutamine, vitamin E and the oral zinc supplement have been the most studied. However, as previously discussed, the provided data are contradictory and do not support their administration for mucositis prevention [17, 81, 113]. Moreover, the promising data are often limited by the small numbers of enrolled patients and/or by the absence of a standardized methodological protocol for compound manufacturing. This is the case of the essential oils of manuka (*Leptospermum scoparium*) and kanuka (*Kunzea ericoides*), whose wound healing, anti-inflammatory, analgesic and anti-micotic and -bacterial properties seem to be effective in OM prevention, as well as the administration of chinese herbal drugs (Indigowood root or *R. algida*), chamomile or aloe vera [113]. Conversely, the systemic topical honey administration is suggested in HNC patients treated with either RT or CT-RT to prevent OM development. [108]. A detailed list of multi target natural agents is reported in Table 1.

#### Physical intervention

Low level application of monochromatic laser and low level laser therapy (LLLT), also called photobiomodulation, applied locally have shown to promote healing of the damage tissue and to inhibit inflammation in animal models [114, 115]. Several clinical studies have further demonstrated the efficacy of LLLT in reducing mucositis severity through the regeneration of damaged-tissue both in patients undergoing chemo-radiotherapy before HSCT [73] that in HNC patients managed with only RT [83]. For those reasons, LLLT application is recommended for OM prevention in the setting of HSCT regimen by MASCC/ISOO guidelines [108]. The same association suggests the use of LLLT application for HNC patients undergoing to CT plus RT or RT only [108]. However, although clinical evidences support the application of this technique in HNC patients, there are in vitro evidences that revealed how LLLT can trigger pro-tumorigenic signaling pathways in tumor cells [114, 116–118]. Accordingly, further studies are mandatory to define a specific guideline for a correct use of LLLT in patients affected by solid tumors. In general, it is recommended to not apply directly low-levels laser impulse on cancer tissue, as well as a strict patient vigilance [83].

MASCC/ISOO guidelines recommend oral cryotherapy use 30 minutes before 5-FU bolus administration, as well as in patients undergoing to autologous HSCT regimen with the presence of high dose of melphalan [108]. The use of ice chips, or ice cubes, or ice lollipops are, indeed, associated with a reduction in 5-FU-mediated OM incidence and severity [73, 81]. Cryotherapy application promotes local vasoconstriction, thus leading to a reduction exposure of mucosa to 5-FU [104].

#### Oral care and probiotics

Although there is no strong evidences of its efficacy for OM prevention, a standardize oral care is suggested by the MASCC / ISOO guidelines [108]. A healthy oral hygiene, indeed, leads to positive benefits, preventing, at least in part, either infections or sepsis events during mucosa ulceration. Besides, a frequent oral cavity examination by oral care experts, before and during anti cancer therapy, could reduce infection risk, as well as could be helpful to reveal earlier mucositis development [105].

Pre-clinical evidences have showed that probiotic administration preserves mucosal intestinal architecture preventing its disaggregation upon damage injury [107]. Accordingly, an active trial aims to test the protective role of Lactobacillus in preventing irinotecan-induced diarrhea (ClinicalTrials.gov Identifier: NCT02819960) [106]. Recently, MASCC / ISOO guidelines have suggested the use of Lactobacillus probiotics to prevent diarrhea in patients with pelvic malignancy treated with RT or RT plus CT [108].

### Mucositis-related symptoms treatment

Mucositis-associated pain severely affects patient quality of life. High-grade mucositis often cause an inadequate food intake; consequently, patients can develop serious nutritional deficiency and need parenteral nutrition. Moreover, about the 15% of them experience a premature therapy termination or a dosage re-modulation, thus affecting survival. Consequently, treatment of pain associated to mucositis is pivotal for cancer patient clinical management.

Analgesics are the most administrated drugs for OM-associated pain control. Morphine is, indeed, recommended by MASCC/ISOO guidelines for OM-associated pain caused by CT and RT treatment in patients undergoing hematopoietic stem cell transplant [119]. Mouth rinses or washes with formula containing morphine are also administrated in HNC patients developing high grade OM. Besides, several “magic” mouthwashes for patients pain control have been formulated. They usually contain anesthetic, antacid and diphenhydramine; sometimes steroids and anti-micotics [83]. A pilot study of 26 HNC patients undergoing to RT plus CT has revealed that patients who managed OM-associated pain with mouthwashes containing 0.2% of morphine complained a shorter duration of severe pain and required lower systemic analgesic administration than that administered to patients who used the magic formulation (lidocaine, magnesium aluminum hydroxide, diphenhydramine) for oral rinses [120]. Topical 0.2% morphine mouthwashes are suggested by MASCC/ISOO guidelines for HNC patients with OM caused by concomitant radio- and chemotherapy [108].

The mucositis study group of MASCC/ISOO has evaluated the effectiveness of additional agents, but the reported data so far did not allow their use for OM-associated pain management. It is the case of chlorhexidine mouthwashes, caphasol re-mineralizing solution rinses or application of topical coating agents [108, 119], such as MuGard whose properties have been evaluated in a multicenter trial reporting only palliative effects [121].

Application of sucralfate enemas, acting as a protective barrier, is suggested for rectal bleeding management caused by RT-induced proctitis; while loperamide, an opioid-receptor agonist, or octreotide, a somatostatin analogue used when loperamide treatment failed, is recommended for diarrhea control in patients undergoing RT plus CT before HSCT [119].

### Biomarker feasibility for oral mucositis development risk assessment and early diagnosis

The opportunity to stratify cancer patients according to their risk to develop mucositis, as well as the possibility to identify mucositis development and its severity in an early phase represents an unmet need for researchers and clinicians. Identification of a standardized biomarker for mucositis assessment and/or early diagnosis might allow, indeed, a precision management of patient, thus reducing hospitalization, therapy termination and dosage re-modulation; ultimately reducing patient management cost. Costs to care a HNC patient who develops high-grade mucositis, indeed, can rise from 2,000 $ up to 6,000 $ in the United States [122].

Cytokines that are released after the chemo- and/or radio-mediated tissue primary injury, act as signal transducers leading to amplification of damage response. Based on their functions and properties, several authors have investigated the feasibility to correlate their levels with mucositis severity and/or mucositis early diagnosis. However, data provided so far are controversial. For instance, TNF-α levels have been found either high [123, 124] or low [125, 126] during RT in different studies, while only one report has shown a significant correlation between TNF-α levels and OM severity [124] (Table 2).

**Table 2:** List of proteins tested as biomarkers of mucositis development and/or severity

Biomarker	Reference	Treatment	Disease	Origin	Levels during treatment	Significant association with OM
**TNF-α**	[123]	RT	HNC	Saliva	High	No
	[124]	RT	HNC	Cytologic smears from oral cavity	High	Association with OM development
	[125]	RT, RT + CT	HNC	Serum	Low	No
	[126]	RT, RT + CT	HNC	Serum	Low	No
**IL-1**	[125]	RT, RT + CT	HNC	Serum	No change	No
	[126]	RT, RT + CT	HNC	Serum	No change	No
**IL-1β**	[124]	RT	HNC	Cytologic smears from oral cavity	High	Association with OM development
**IL-6**	[123]	RT	HNC	Saliva	High	Association with OM severity
	[125]	RT, RT + CT	HNC	Serum	High	Association with OM severity
**IL-8**	[123]	RT	HNC	Saliva	High	No
	[125]	RT, RT + CT	HNC	Serum	High	No
**IL-10**	[123]	RT	HNC	Saliva	High	Association with OM severity
	[125]	RT, RT + CT	HNC	Serum	No change	No
**TGF-β**	[127, 128]	RT + CT	HNC	Plasma	High	Association with OM severity
**EGF**	[2, 129, 130]	RT	HNC	Saliva	Low	Association with OM development
	[131]	RT	HNC	Saliva	Low	Association with OM development
**CRP**	[132]	RT	HNC	Blood	High	Association with OM severity
	[133]	RT	HNC	Blood	High	Not assessed
	[134]	RT, RT + CT	HNC	Blood	High	Not assessed
	[135]	RT + CT	HNC	Blood	High	Association with OM severity only at initial week
**ESR**	[132]	RT	HNC	Blood	High	No
	[134]	RT, RT + CT	HNC	Blood	High	Not assessed
	[135]	RT + CT	HNC	Blood	High	No
**GSH**	[136]	RT	Oral cavity cancer	Plasma	Measured before RT	GSH baseline levels associate with OM development
	[137]	RT	HNC	Plasma	No change	No
**DNA DSB (γ-H2AX)**	[138]	RT	HNC	Peripheral blood lymphocytes	High	Association with OM severity
	[139]	RT, RT + CT	HNC	Peripheral blood lymphocytes	High	No

The same controversial results have been provided by studies that correlated IL-10 and IL-1 levels and OM in HNC patients undergoing to CT or/and RT [123, 125, 126] (Table 2). Further evidences are mandatory to confirm the correlation between high-grade mucositis development and IL-6 or IL-1β levels in RT treated patients [123–125] (Table 2).

High levels of TGF-β have been found in the plasma of patients experiencing a RT-induced severe toxicity [127, 128]. However, while TGF-β concentration increased in response to RT, it did not correlate with risk of mucositis development [127, 128]. Conversely, plasma levels of the epidermal growth factor (EGF) negatively correlated with risk of OM development. Indeed, HNC patients with low levels of EGF before treatment exhibited a major risk to develop OM during RT [129–131] (Table 2).

The feasibility to use markers of the so call “inflammatory acute phase” as mucositis biomarkers has been also evaluated. The C-reactive protein (CRP) and the erythrocyte sedimentation rate (ESR) are two important markers which assessment is routinely done to diagnose the presence of an inflammation process through blood test. Their levels have been found high in the blood of patients at the end of RT [132–135]. Notably, Ki et al., have found a correlation between the increase in CRP, but not ESR, levels and mucositis progression [132], while Chethana et al., have found a correlation between OM and CRP levels only in the first weeks of treatment [135] (Table 2).

Sporadic studies have evaluated a putative correlation between the OM development and/or severity and the levels of different proteins usually involved in processes such as apoptosis, ROS scavenger, adhesion and structural proteins. In particular, high levels of p53 [124], BPI Fold Containing Family A Member 1 (BPIFA-1) [140], Intercellular Adhesion Molecule 1 (ICAM-1), E-selectin, Lymphocyte function-associated antigen 1 (LFA-1) and macrophage integrin (Mac-1) have been found in presence of high-grade mucositis [141]. Conversely, low levels of pro-apoptotic proteins such as the B-cell lymphoma 2 (BCL-2) and the induced myeloid leukemia cell differentiation protein (Mcl-1) [124], as well as low levels of antioxidant glutathione GSH have been reported [136]. Notably, meta-analysis conducted by Normando and colleagues have collected and analyzed this evidence without finding a strong correlation between the levels of the previous cited proteins and OM process [142]. The heterogeneity of the analyzed studies and the low numbers of enrolled patients, indeed, do not allow their dosage for mucositis prediction [142]. The specificity of these proteins for OM assessment should be further investigated.

As reported above, radiotherapy directly injures mucosa provoking double strand breaks (DSBs) of DNA. Accordingly, a reduction in the activities of key proteins involved in the DNA repair could correlate with mucositis incidence and/or with its severity. Among them, the histone protein γ-H2AX levels correlated with radiotherapy-induced toxicities, such as oral mucositis [138, 139] (Table 2). Moreover, the efficiency in DSBs repair can change among patients basing on the presence of single-nucleotide polymorphisms (SNPs) in the sequence of genes involved in this process. Indeed, presence of specific SNPs modulates activities of the related translated protein [143]. It is the case of some SNPs harbored in genes such as XRCC1, XRCC3 and RAD51, which activities are critical during the DNA repair. Their presence has been related to an increased risk to develop radiotherapy mediated toxicities [144–146]. However, the correlation with OM is not so strong [142]; further studies are needed to support their assessment for OM development.

In 2017, Gutierrez-Camino and colleagues have identified a significant correlation between the presence of SNP rs10505168 in the sequence of miR-2053 and an increased risk to develop OM in children undergoing methotrexate treatment for pediatric acute lymphoblastic leukemia (ALL) treatment [147].

Recently, Laheji and colleagues have undertaken oral microbioma analysis from patients undergoing to CT plus RT treatment before autologous hematopoietic stem cell transplantation and revealed that patients who did not develop an ulcerative OM exhibited more resilient microbioma than those patients developing an ulcerative OM [148].

As previously described, most of the so far tested biomarkers require further studies to be validated either for mucositis diagnosis or for mucositis severity prediction. A standardized protocol for their assessment, as well as strict criteria of patient enrolment are mandatory to clinically validate their use as biomarkers. However, these aspects may not be enough, the main bias, indeed, is that none of them is a specific marker that characterizes mucositis process. They are, indeed, produced as consequence of different stresses. That aspect could compromise their feasibility as mucositis biomarkers. During antineoplastic treatment, the whole organism undergoes to several stresses that could either hide or mimic mucositis process. It could be helpful to find a marker that specifically identifies mucositis process development. Non-coding RNAs have been found to be more lineage-specific than protein coding genes, unveiling how their different expression might specifically determine cell phenotype [149–151]. Accordingly, this may not be foolish the research of non-coding RNAs that are specifically involved in mucositis process, whose assessment could be used as mucositis biomarker.

## Conclusions

The advance in cancer therapies has significantly improved survival of patients. However, while therapies become increasingly effective, only few valid options are available for antineoplastic therapy-induced oral mucositis treatment or prevention; which often cause either treatment premature termination or re-modulation. This also increases hospitalization with a consequently increase of cost for public health and reduction of the patient quality of life. The opportunity to identify patient susceptibility to develop OM, through an accessible and non-invasive test assessing of OM-related specific biomarkers expression, might allow to design a personalized targeted treatment. This might also allow testing the prevention activity of new agents in a high-risk sub population, thus improving the significance of the clinical outcome.

Nowadays, oral mucositis still remains an underestimated side effect of anticancer therapy. The synergistic efforts of basic, translational and clinical scientists is strongly required to improve cancer patients quality of life and, consequently, reduce their management cost.

## Data Availability

Not applicable.

## Abbreviations

CT: Chemotherapy
RT: radiotherapy
GIM: gastro-instestinal mucositis
OM: oral mucositis
HSCT: hemopoietic stem cell transplantation
HNC: head and neck cancer
5-FU: 5-Fluoruracil
WHO: World Health Organization
OMAS: Oral Mucositis Assessment Scale
ECOG: Eastern Cooperative Oncology Group
NCI: National Cancer Institute
JNK: Common Terminology Criteria for Adverse kinase
NRF2: erythroid 2-related factor 2
AP1: activating protein-1
MMP: metalloproteinase
MAPK: mitogen activated protein kinase
TGF: tumor growth factor
mIAS: mTOR-inhibitor-associated stomatitis
RCC: renal cell carcinoma
EGFR: epidermal growth factor
TKi: tyrosine kinase inhibitor
T-DM-1: ado-trastuzumab emtansine
CDK4/6: cyclin-dependent kinase 4/6 inhibitors
FDA: Food and Drug Administration
VEGFR: vascular endothelial growth factor receptor
irAE: immunotherapy-related adverse event
PDL1: anti-programmed death ligand 1
PD1: anti-programmeddeath 1
CTLA4: cytotoxic T lymphocyte-associated antigen 4
KGF-1: keratinocyte growth factor 1
EMA: European Medical Agency
NAC: N-acetyl-cysteine
SOD: superoxide dismutase
MASCC/ISOO: Multinational Association of Supportive Care in Cancer and International Society of Oral Oncology
LLLT: low-levels laser therapy
CRP: C-reactive protein
ESR: erythrocyte sedimentation rate
BPIFA-1: BPI Fold Containing Family A Member 1
ICAM-1: Intercellular Adhesion Molecule 1
LFA-1: lymphocyte function associated antigen 1
Mac-1: macrophage integrin
BCL-2: B-cell lymphoma 2
Mcl-1: induced myeloid leukemia cell differentiation protein
DSBs: double strand breaks
SNPs: single-nucleotide polymorphisms
ALL: acute lymphoblastic leukemia
